# WNV and SLEV coinfection in avian and mosquito hosts: impact on viremia, antibody responses, and vector competence

**DOI:** 10.1128/jvi.01041-24

**Published:** 2024-09-26

**Authors:** Emily N. Gallichotte, Emily A. Fitzmeyer, Landon Williams, Mark Cole Spangler, Angela M. Bosco-Lauth, Gregory D. Ebel

**Affiliations:** 1 Department of Microbiology, Immunology and Pathology, Colorado State University, Fort Collins, Colorado, USA; 2 Department of Biomedical Sciences, Colorado State University, Fort Collins, Colorado, USA; Emory University School of Medicine, Atlanta, Georgia, USA

**Keywords:** arbovirus, coinfection, vector competence, flavivirus

## Abstract

West Nile virus (WNV) and St. Louis encephalitis virus (SLEV) are closely related flaviviruses that can cause encephalitis in humans and related diseases in animals. In nature, both are transmitted by *Culex*, with wild birds, including jays, sparrows, and robins, serving as vertebrate hosts. WNV and SLEV circulate in the same environments and have recently caused concurrent disease outbreaks in humans. The extent that coinfection of mosquitoes or birds may alter transmission dynamics, however, is not well characterized. We therefore sought to determine if coinfection alters infection kinetics and virus levels in birds and infection rates in mosquitoes. Accordingly, American robins (*Turdus migratorius*), two species of mosquitoes, and vertebrate and invertebrate cells were infected with WNV and/or SLEV to assess how simultaneous exposure may alter infection outcomes. There was variable impact of coinfection in vertebrate cells, with some evidence that SLEV can suppress WNV replication. However, robins had comparable viremia and antibody responses regardless of coinfection. Conversely, in *Culex* cells and mosquitoes, we saw a minimal impact of simultaneous exposure to both viruses on replication, with comparable infection, dissemination, and transmission rates in singly infected and coinfected mosquitoes. Importantly, while WNV and SLEV levels in coinfected mosquito midguts were positively correlated, we saw no correlation between them in salivary glands and saliva. These results reveal that while coinfection can occur in both avian and mosquito hosts, the viruses minimally impact one another. The potential for coinfection to alter virus population structure or the likelihood of rare genotypes emerging remains unknown.

**IMPORTANCE:**

West Nile virus (WNV) and St. Louis encephalitis virus (SLEV) are closely related viruses that are transmitted by the same mosquitoes and infect the same birds in nature. Both viruses circulate in the same regions and have caused concurrent outbreaks in humans. It is possible that mosquitoes, birds, and/or humans could be infected with both WNV and SLEV simultaneously, as has been observed with Zika, chikungunya, and dengue viruses. To study the impact of coinfection, we experimentally infected vertebrate and invertebrate cells, American robins, and two *Culex* species with WNV and/or SLEV. Robins were efficiently coinfected, with no impact of coinfection on virus levels or immune response. Similarly, in mosquitoes, coinfection did not impact infection rates, and mosquitoes could transmit both WNV and SLEV together. These results reveal that WNV and SLEV coinfection in birds and mosquitoes can occur in nature, which may impact public health and human disease risk.

## INTRODUCTION

West Nile virus (WNV) and St. Louis Encephalitis virus (SLEV) are closely related flaviviruses that can cause encephalitic disease in humans (1). They are genetically and antigenically related, with single-stranded positive sense ~11 kb RNA genomes (1). Both viruses are maintained in nature in enzootic cycles with birds as hosts and *Culex* mosquitoes, mainly *Culex pipiens*, *Culex quinquefasciatus*, and *Culex tarsalis* as vectors (2–6). Infected mosquitoes occasionally transmit these viruses to humans, horses, and other mammals, which serve as dead-end hosts and do not maintain the infection cycle in nature (4). Many birds have been implicated in the natural transmission cycles for both WNV and SLEV, including American robins, house sparrows, and American crows (7–11). Additionally, experimental infections demonstrate there are many bird species susceptible to both viruses, including jays, finches, and warblers (12–14). WNV and SLEV are clinically and ecologically similar viruses that pose ongoing threats to humans and animals.

SLEV was first detected in the United States in 1933 in St. Louis, MO (15). SLEV has a broad geographic range covering North and South America; however, the majority of human cases are reported in the United States (1). In 1999, WNV was introduced in New York City and, in the years following, spread throughout the country displacing SLEV in many places (16, 17). Nonetheless, SLEV continued to circulate, causing human disease in South America, primarily in Argentina, Brazil, and Peru (18). SLEV was again detected in the western United States in 2014, and phylogenetic analyses revealed that the circulating viruses were most similar to strains from a human outbreak in Argentina in 2005 and suggested a reintroduction of the virus and not reemergence of latent/low levels of virus in the environment (18). WNV and SLEV currently cocirculate in regions of the United States and were simultaneously detected in chickens and mosquito pools in the Coachella Valley of Southern California in 2015, where SLEV had not been detected since 2003 (19). Comprehensive analyses from 2015 to 2020 using sentinel chicken and mosquito surveillance showed that both viruses were widely prevalent and cocirculated throughout California during that time (20, 21). Additionally, from January to July of 2015 in Arizona, there was a large increase in the number of human SLEV cases (21, compared with just 1 during all of 2010–2014) (22). There were also WNV human cases during this same time period (75 cases) (22), and many mosquito pools tested positive for SLEV and WNV (23). While there are no documented cases of coinfection in either humans [antigenic cross-reactivity make serologically distinguishing past WNV vs SLEV infection challenging (24)] or mosquitoes, it is clear the viruses cocirculate in the same geographic regions and cause concurrent human disease. It is therefore possible that undetected coinfections have occurred in humans, mosquitoes, and/or birds.

Coinfection of arboviruses in humans and other mammals is a growing concern due to the continued emergence and reemergence of many of these viruses (25). Mathematical modeling suggests coinfections are most likely to occur in tropical climates, where temperatures are most favorable to cocirculation of multiple viruses (26). In nature, multiple bird species have been found coinfected with WNV and Usutu virus (USUV) (another closely related encephalitic flavivirus) (27, 28). Although uncommon, there are documented cases of humans infected by dengue virus (DENV) and chikungunya virus (CHIKV), DENV and Zika virus (ZIKV), CHIKV and ZIKV, WNV and USUV, and even simultaneous coinfections with DENV, CHIKV, and ZIKV (29–33). There are also documented cases of horses coinfected with WNV and Eastern equine encephalitis virus and WNV and Sindbis virus (34–36). Experimental studies have shown that mosquitoes may become coinfected and can simultaneously transmit multiple viruses (37–43). Importantly, sequential infection of *Cx. quinquefasciatus* mosquitoes with WNV and SLEV found that prior exposure to one virus lowered infection and dissemination rates with the second virus; however, simultaneous coinfection was not performed (44).

Therefore, we hypothesized that WNV and SLEV coinfection would have minimal impact on infection in multiple hosts. We investigated the impact of WNV and SLEV coinfection in vertebrate and invertebrate cells, American robin viremia and antibody responses, and *Culex* mosquito infection, transmission, and dissemination rates and virus levels. Our data reveal that while cells, birds, and mosquitoes can be efficiently coinfected with both viruses after simultaneous exposure, frequently (especially in mosquitoes and mosquito cells), the viruses appear to have no impact on one another. These results align with many other studies of experimentally coinfected mosquitoes, finding that overall infection rates and virus levels are similar in coinfected mosquitoes as compared with those individually infected (37–40).

## RESULTS

### Impact of WNV and SLEV coinfection in vertebrate cells

**Fig 1:**
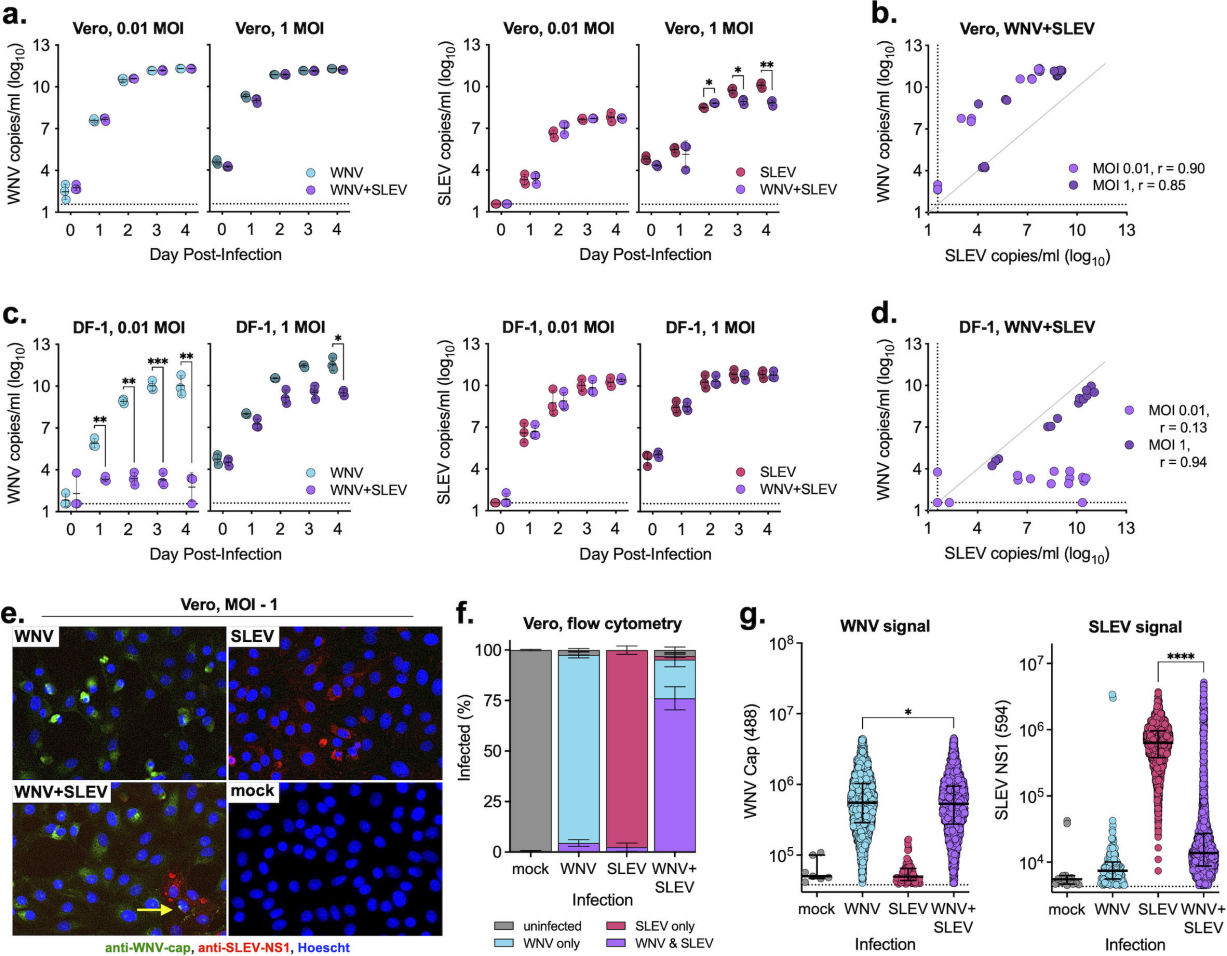
WNV and SLEV coinfection in vertebrate cells. (**a and b**) Vero (African green monkey) and (**c and d**) DF-1 (chicken) cells were infected at two MOIs individually or coinfected with WNV and SLEV; supernatant was sampled daily and tested for viral RNA as measured by quantitative real-time PCR (qRT-PCR) (performed in biological triplicate, mean ± standard deviation). Two-way analysis of variance (ANOVA) with Šídák’s multiple comparison test, *P < 0.05, **P < 0.01, and ***P < 0.0005. Relationship between WNV and SLEV in the coinfected (**b**) Vero and (**d**) DF-1 cells. Spearman *r* noted in figures. (**e**) Vero cells were individually infected or coinfected (MOI = 1) and, after 3 days, fixed and stained for WNV and SLEV viral protein and (**e**) imaged for confocal microscopy and (**f and g**) flow cytometry. (**f**) Percentages of uninfected, singly infected, and coinfected cells were determined for each infection conditions (performed in biological triplicate, mean ± standard deviation). (**g**) WNV capsid 488 and SLEV NS1 594-positive populations were randomly downsized to 10% of the original number of cells, and fluorescent intensity was compared (median ± interquartile range). One-way ANOVA with Tukey’s multiple comparison test, *P < 0.05 and ****P < 0.0001. Flow cytometry plots and gating are shown in Fig. S2. Dashed lines represent limits of detection.

To evaluate the impact of coinfection on virus replication in vertebrate cells, we infected Vero (African green monkey, kidney) and DF-1 (chicken, fibroblast) cells with WNV, SLEV, or both and measured the levels of extracellular virus (Fig. 1). We found that in Vero cells, coinfection has minimal impact on the level of extracellular WNV at either a low (0.01) or high (1) multiplicity of infection (MOI); however, at a high MOI, WNV significantly reduced SLEV RNA (Fig. 1a). The relationship between the levels of WNV and SLEV produced by coinfected cells was highly correlated regardless of MOI (Spearman *r* > 0.8) (Fig. 1b). In DF-1 cells, SLEV coinfection significantly suppressed WNV replication at low MOIs; however, coinfection did not impact SLEV replication (Fig. 1c). The relationship between WNV and SLEV and coinfected cells varied depending on MOI, with a more dramatic suppression seen at a low MOI (Fig. 1d).

Due to the possible interaction between viruses in coinfection conditions, we determined if individual cells were coinfected using fluorescent microscopy of Vero cells infected at high MOIs (1 and 5) at 3 days post-infection (Fig. 1e; Fig. S1). We saw high infection rates in the single infection conditions for both viruses and in coexposed cells, individual cells that appeared to be infected with both WNV and SLEV (Fig. 1e, yellow arrow). Interestingly, we also saw a reduction in total number of cells infected with SLEV in coinfection conditions as compared with SLEV alone (Fig. 1e). To quantitively measure the percentages of single and coinfected cells and levels of intracellular viral protein, we performed flow cytometry on Vero cells infected at an MOI of 1 at 3 days post-infection (Fig. 1f and g; Fig. S2). Consonant with microscopy results, we found a high level of infected cells in single infection conditions (>90%); however, there was a reduced percentage of SLEV-infected cells under coinfection conditions (~75% coinfected and ~2% SLEV single infection) (Fig. 1f). This reduction in SLEV-infected cells likely explains the reduced levels of SLEV extracellular virus when WNV is present (Fig. 1a; Fig. S2a). Importantly, we noticed a dramatic shift in the SLEV intracellular protein level in coexposed cells as compared with those infected with just SLEV (Fig. S2f and g). While levels of WNV intracellular protein were slightly decreased in coexposed cells compared with singly infected cells, levels of intracellular SLEV were significantly decreased (*P* < 0.0001) in cells when WNV was present (Fig. 1g).

### Coinfection of American robins

**Fig 2:**
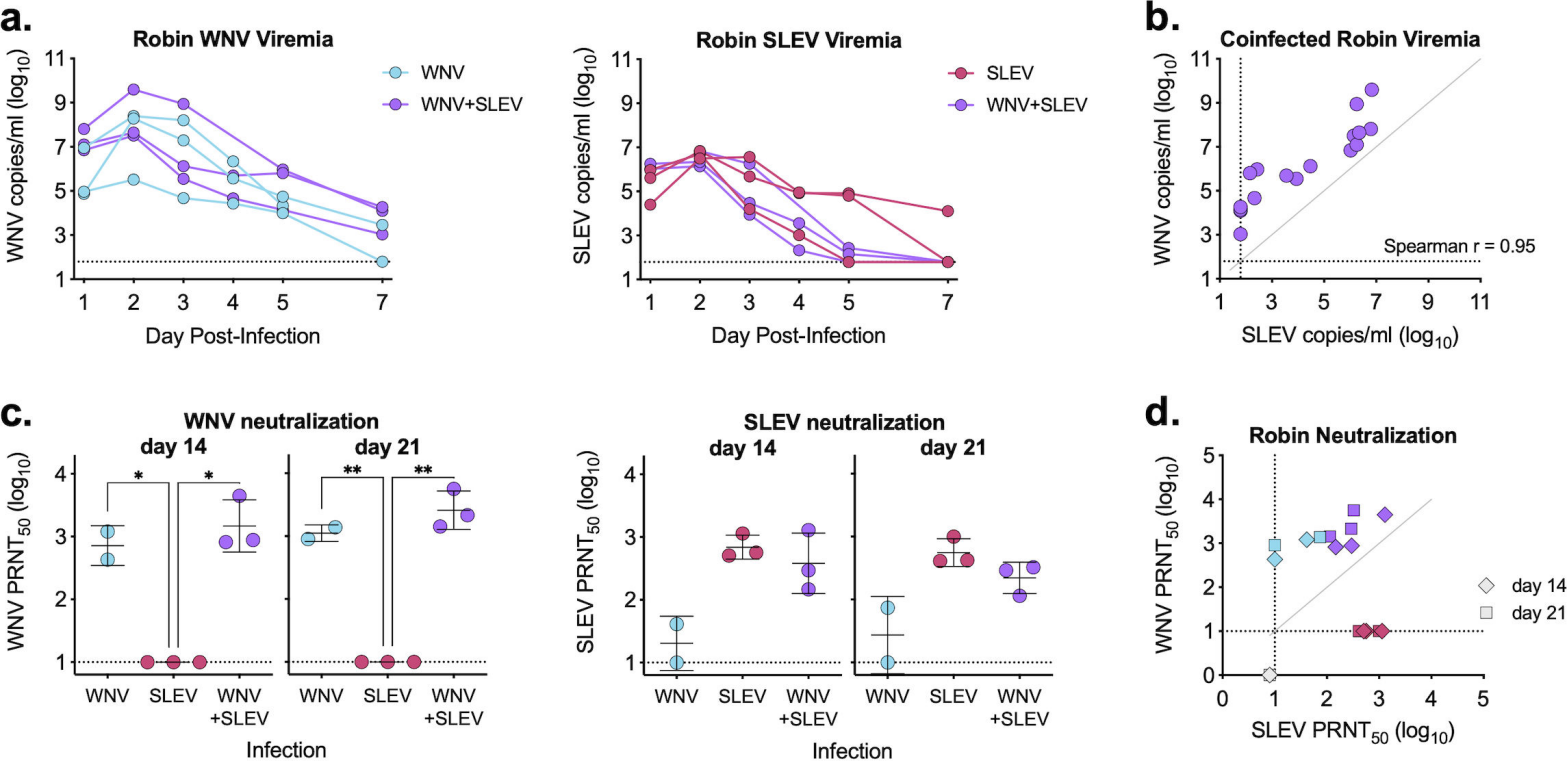
WNV and SLEV coinfection of American robins. (**a**) American robins were individually infected or coinfected with WNV and/or SLEV; blood was collected through day 7 and analyzed for viral RNA as measured by qRT-PCR (performed in biological triplicate). No comparisons were significant by two-way ANOVA with Šídák’s multiple comparison test (*P* > 0.05). (**b**) Relationship between WNV and SLEV in coinfected American robins, Spearman *r* noted in figure. (**c**) Serum collected on days 14 and 21 was analyzed for neutralization against both WNV and SLEV using a standard plaque reduction neutralization test. PRNT_50_ (serum dilution factor required to neutralize 50% of virus) are plotted (mean ± standard deviation). Samples with no neutralization are plotted at half the limit of detection. Two-way ANOVA with Tukey’s multiple comparison test (*P < 0.05). (**d**) Relationship between WNV and SLEV neutralization titers (diamond—day 14, square—day 21). Dashed lines represent limits of detection.

We next infected American robins with either WNV or SLEV individually (10^4^ PFU) or in combination (10^4^ PFU WNV and 10^4^ PFU SLEV). Blood was collected, and levels of viral RNA were measured via quantitative real-time PCR (qRT-PCR). We found that coinfection of WNV and SLEV had no impact on viremia compared with single infection for either virus (Fig. 2a). All birds reached peak viremia on day 2 post-infection; however, peak viremia was higher in WNV-infected birds (~10^8^ genome copies/mL) as compared with SLEV-infected birds (~4 × 10^6^ genome copies/mL) (Fig. 2a). When comparing levels of WNV and SLEV in coinfected birds at all times post-infection, we saw a strong positive relationship between the two viruses (Spearman *r* = 0.95) (Fig. 2b), suggesting the total virus level is bird specific. We collected serum on days 14 and 21 post-infection to analyze neutralizing antibody responses to both WNV and SLEV (Fig. 2c). We found that WNV-infected and coinfected birds generated comparable WNV-neutralizing antibodies (Fig. 2c). Similarly, serum from both SLEV individually infected and coinfected birds neutralized SLEV (Fig. 2c). There was minimal cross-neutralization of the heterologous virus in both single infection groups (Fig. 2c). Coinfected birds generated higher neutralizing antibody titers to WNV than SLEV (Fig. 2d), likely due to the higher level of WNV viremia. Results were comparable when using an 80% neutralization threshold (Fig. S3).

### Coinfection of mosquito cells

**Fig 3:**
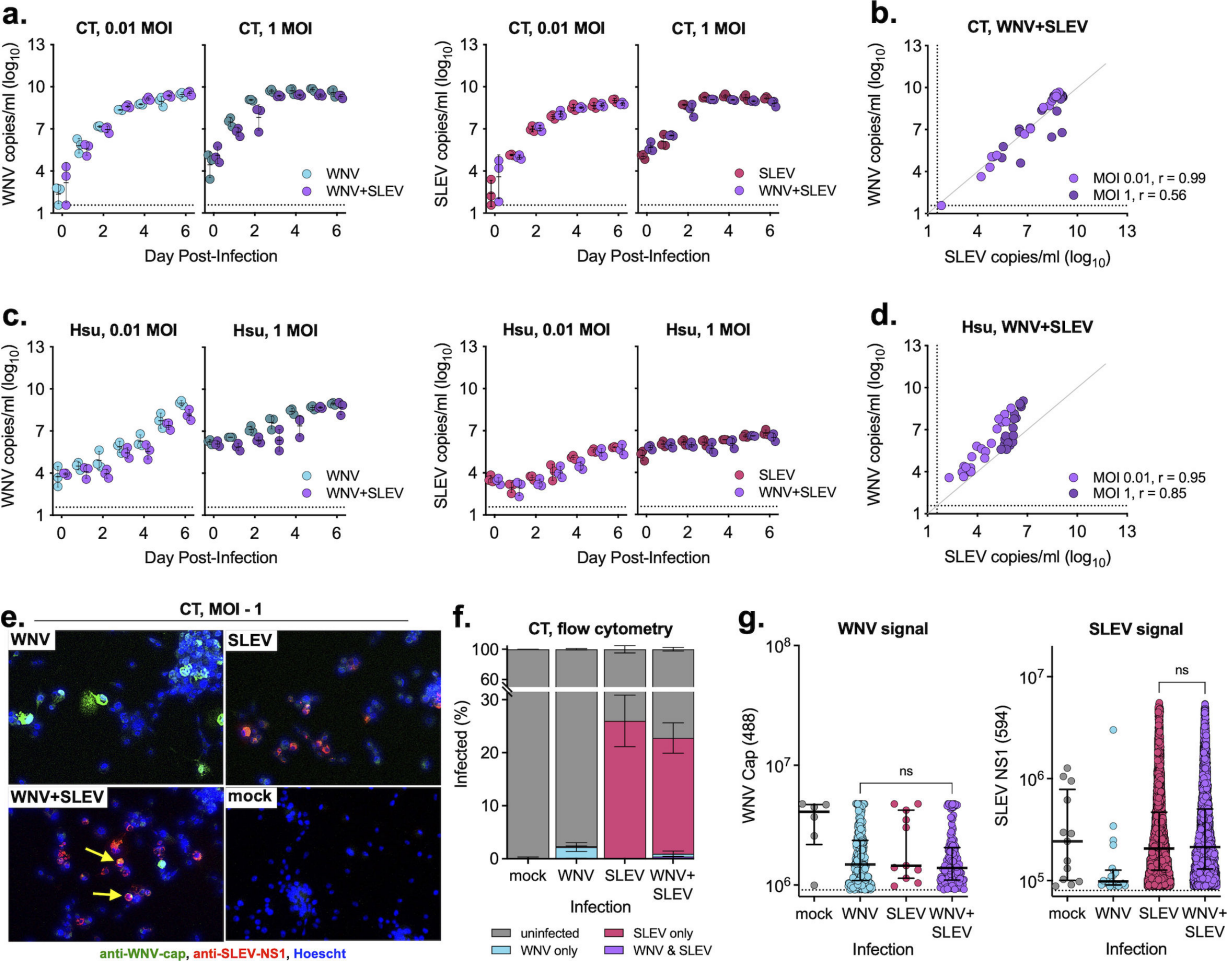
WNV and SLEV coinfection in mosquito cells. (**a and b**) CT (*Cx. tarsalis*) and (**c and d**) Hsu (*Cx. quinquefasciatus*) cells were infected at two MOIs individually or coinfected with WNV and SLEV; supernatant was sampled daily and tested for viral RNA as measured by qRT-PCR (performed in biological triplicate, mean ± standard deviation). No comparisons were significant with a two-way ANOVA with Šídák’s multiple comparison test, *P* > 0.05. Relationship between WNV and SLEV in the coinfected (**b**) CT and (**d**) Hsu cells. Spearman *r* noted in figures. (**e and f**) Cells were individually infected or coinfected and after 5 days fixed and stained for WNV and SLEV viral protein and (**e**) imaged for confocal microscopy and (**f and g**) flow cytometry. (**f**) Percentages of uninfected, singly infected, and coinfected cells were determined for each infection conditions (performed in biological triplicate, mean ± standard deviation). (**g**) Fluorescent intensity rates of WNV capsid 488 and SLEV NS1 594-positive populations were compared (median ± interquartile range). Comparisons were not significant with a one-way ANOVA with Tukey’s multiple comparison test. Flow cytometry plots and gating are shown in Fig. S2. Dashed lines represent limits of detection.

We next sought to measure the impact of coinfection in two *Culex* cell lines. We infected CT (*Cx. tarsalis*) and Hsu (*Cx. quinquefasciatus*) cells with WNV, SLEV, or both in combination and measured extracellular virus via qRT-PCR. We found that in CT cells, coinfection had no impact on viral replication for either virus (Fig. 3a) and that there was a strong positive relationship between levels of WNV and SLEV in coinfected cells regardless of MOI (Fig. 3b). In Hsu cells, there was a subtle, non-significant decrease of WNV in SLEV-coinfected cells as compared with WNV individually infected cells (Fig. 3c), with a strong relationship between levels of WNV and SLEV in coinfected cells (Spearman *r* > 0.8) (Fig. 3d). Despite limited evidence for interaction between the viruses, we performed microscopy of CT cells infected at high MOIs (1 and 5) at 5 days post-infection (Fig. 3E; Fig. S4). Unlike Vero cells, we saw much lower levels of infection in both single and coinfection conditions; however, there were rare instances of coinfected cells (<1%) (Fig. 3e and f). Flow cytometry revealed low infection rates in singly infected cells (~2% WNV infected, ~26% SLEV infected), which were only minimally altered in coinfection conditions (Fig. 3f). Unlike Vero cells, levels of intracellular WNV and SLEV protein were similar across infection conditions and cell populations (Fig. 3g; Fig. S5f and g).

### Coinfection does not alter mosquito infection rates or levels

**Fig 4:**
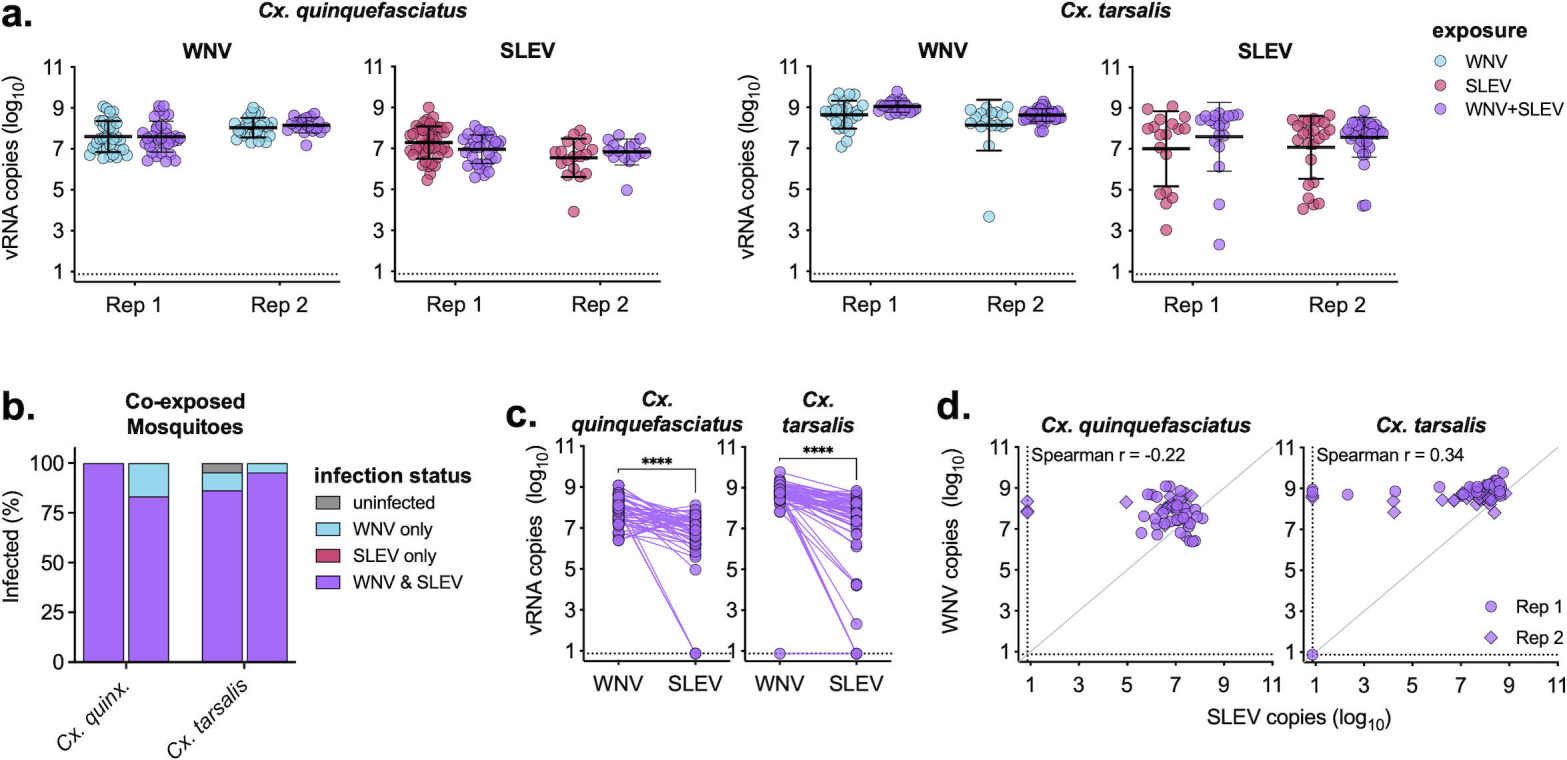
*Culex* mosquitoes are efficiently coinfected with WNV and SLEV. (**a**) *Cx. quinquefasciatus* and *Cx. tarsalis* were exposed to bloodmeals containing either WNV and SLEV individually or in combination. On day 7, whole bodies were processed, RNA extracted, and measured for viral RNA via qRT-PCR. Experiment performed in biological duplicate (*n* = 18–45 mosquitoes/group, mean ± standard deviation). Only samples with detectable virus are plotted. Virus levels of single infection vs coinfection (e.g., WNV levels in WNV infection vs WNV + SLEV infection) were not significant with a two-way ANOVA with Šidák’s multiple comparison test (*P* > 0.05). (**b**) Percentage of coexposed mosquitoes that were uninfected, infected with WNV only, infected with SLEV only, or coinfected with WNV and SLEV (both replicates shown). (**c**) Comparison between levels of WNV and SLEV in coexposed mosquitoes (both replicates combined). Paired t-test, ****P < 0.0001. (**d**) Relationship between WNV and SLEV in coexposed mosquitoes (circle—replicate 1, diamond—replicate 2). Spearman *r* noted in figures. Dashed lines represent limits of detection.

Despite minimal impact of coinfection on WNV and SLEV viral replication in *Culex* cell lines, we wanted to determine the impact of coinfection in two *Culex* spp. mosquitoes. *Cx. quinquefasciatus* and *Cx. tarsalis* were fed infectious bloodmeals containing either WNV or SLEV individually or in combination (Table 1). On day 7 post-exposure, mosquito bodies were collected and analyzed for WNV and SLEV infection via qRT-PCR. Both viruses had high infection rates in both species (>75%), and coinfection had minimal impact on total infection rates (Table 1). We next compared the levels of WNV and SLEV RNA in individually and coinfected mosquitoes and found no significant differences (*P* > 0.05) for either of the two species (Fig. 4a). Of the coexposed mosquitoes, the majority (>80%) were coinfected with both WNV and SLEV, with a small fraction uninfected or only infected with WNV (Fig. 4b). There was significantly more WNV than SLEV in coexposed mosquitoes in both species (*P* < 0.0001) (Fig. 4c); however, there was no relationship between vRNA levels (Spearman *r* < 0.4) for either of the species, suggesting levels of each virus within a mosquito were unrelated (Fig. 4d).

**TABLE 1:** Infection rates of *Culex* mosquitoes infected with WNV and SLEV^*a*^

Species	Infection	Replicate 1		Replicate 2	
		WNV	SLEV	WNV	SLEV
*Cx. quinquefasciatus*	WNV	100% (34/34)	–	96% (27/28)	–
	SLEV	–	100% (45/45)	–	86% (18/21)
	WNV + SLEV	100% (33/33)	100% (33/33)	100% (18/18)	83% (15/18)
*Cx. tarsalis*	WNV	100% (24/24)	–	100% (19/19)	–
	SLEV	–	75% (18/24)	–	90% (26/29)
	WNV + SLEV	95% (21/22)	86% (19/22)	100% (44/44)	95% (41/43)

^*a*^ Infection rates were determined by detection of viral RNA via qRT-PCR. There were no significant differences between single infection and coinfection for each virus, mosquito species, and replicate using a chi-square test. – indicates samples that were not tested.

### Impact of coinfection on infection, dissemination, and transmission rates

We next infected *Cx. quinquefasciatus* mosquitoes with WNV and SLEV individually or in combination and on days 7 and 14, dissected midguts, legs and wings, salivary glands, and saliva and performed qRT-PCR to measure infection and levels of viral RNA (Table 2). At both days 7 and 14, midgut infection rates for both viruses were high (>90%) regardless of infection (single vs coinfected). Dissemination of WNV from the midgut into the legs and wings was higher than that of SLEV, but neither was impacted by coinfection (Table 2). We saw a significant decrease in salivary gland infection at day 7 in WNV and SLEV-coinfected mosquitoes as compared with WNV alone (41% compared with 72%); however, by day 14 post-infection, there was no difference (81% positive salivary glands for both) (Table 2). Coinfection decreased the rate of WNV-positive saliva samples (44% and 22%, for WNV single and WNV + SLEV coinfection, respectively), but when accounting for salivary gland infection rates, the transmission rates were comparable [61% (14/23) and 54% (7/13) for WNV single infection and coinfection, respectively] (Table 2). Despite SLEV having lower overall infection rates compared with WNV, coinfection had minimal impact on SLEV infection at either day 7 or 14 in any of the samples studied (Table 2).

**TABLE 2:** Infection rates of *Cx. quinquefasciatus* midguts, legs and wings, salivary glands, and saliva infected with WNV and SLEV^*a*^

Sample type	Infection	Day 7		Day 14	
		WNV	SLEV	WNV	SLEV
Midgut	WNV	100% (32/32)	–	100% (32/32)	–
	SLEV	–	97% (30/31)	–	97% (29/30)
	WNV + SLEV	94% (29/31)	90% (28/31)	100% (32/32)	97% (31/32)
Legs and wings	WNV	71% (22/31)	–	97% (31/32)	–
	SLEV	–	16% (5/32)	–	78% (25/32)
	WNV + SLEV	59% (19/32)	19% (6/32)	97% (31/32)	66% (21/32)
Salivary glands	WNV	72% (23/32)	–	81% (26/32)	–
	SLEV	–	16% (5/32)	–	38% (12/32)
	WNV + SLEV	41% (13/32)*	16% (5/32)	81% (26/32)	41% (13/32)
Saliva	WNV	44% (14/32)	–	44% (14/32)	–
	SLEV	–	3% (1/32)	–	13% (4/32)
	WNV + SLEV	22% (7/32)	3% (1/32)	60% (19/32)	19% (6/32)

^*a*^ Infection rates were determined by detection of viral RNA via qRT-PCR. Significant differences between single and coinfection for each virus, sample, and time point using a chi-square test are shown (*P < 0.05). – indicates samples that were not tested.

### Impact of coinfection on midgut, leg and wing, salivary gland, and saliva virus levels

**Fig 5:**
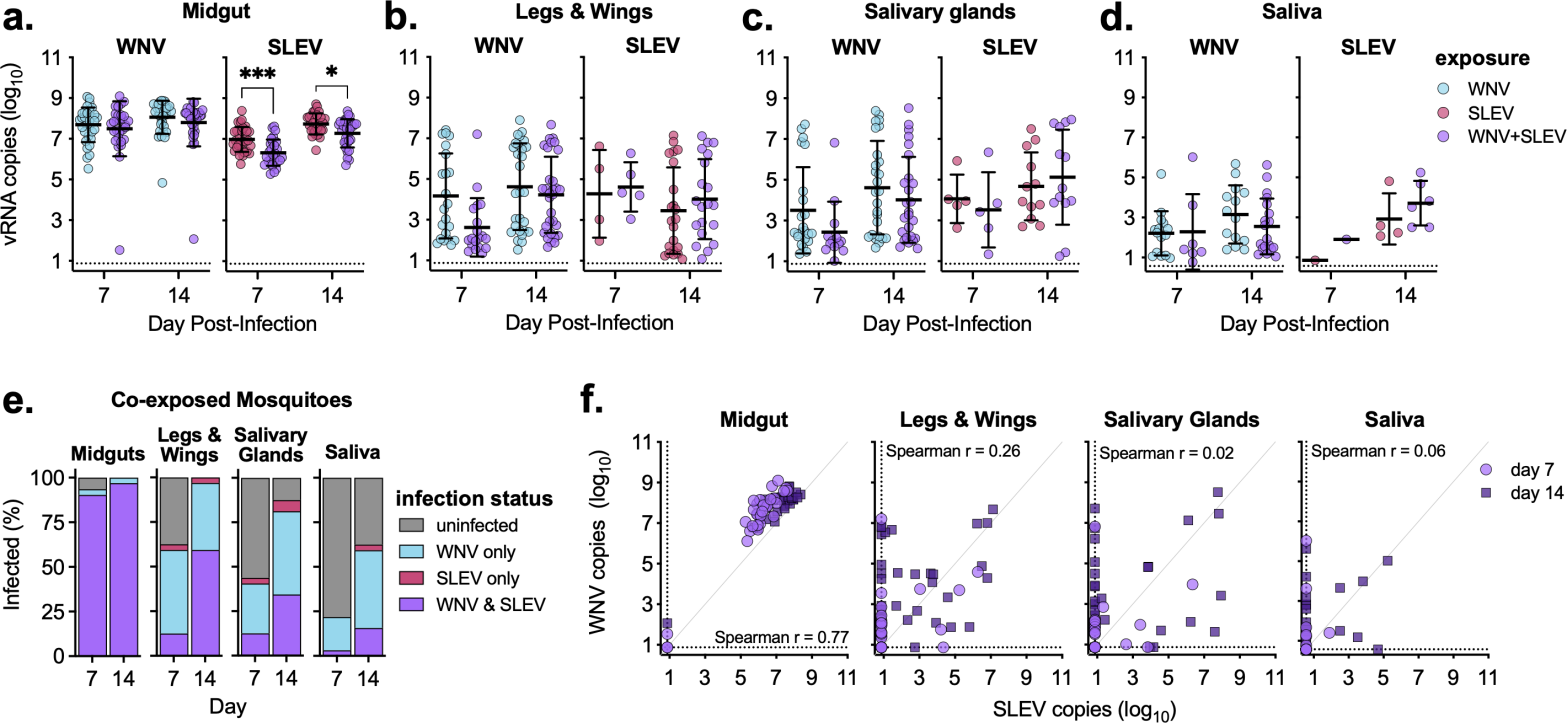
WNV and SLEV coinfection does not alter *Cx. quinquefasciatus* tissue virus levels. (**a–d**) *Cx. quinquefasciatus* mosquitoes were exposed to an infectious bloodmeal containing WNV and SLEV individually or in combination, and on days 7 and 14 post-exposure, mosquito (**a**) midguts, (**b**) legs and wings, (**c**) salivary glands, and (**d**) saliva were collected and analyzed for viral RNA via qRT-PCR (*n* = 30–32 mosquitoes/group, mean ± standard deviation). Only samples with detectable virus are plotted. Ordinary one-way ANOVA with Tukey’s multiple comparison test, *P < 0.05 and ***P < 0.001. (**e**) Percentage of coexposed mosquito midguts, salivary glands, and saliva that were uninfected, infected with WNV only, infected with SLEV only, or coinfected with WNV and SLEV. (**f**) Relationship between WNV and SLEV in coexposed mosquito midguts, legs and wings, salivary glands, and saliva (circle—day 7, square—day 14). Spearman *r* noted in figures. Dashed lines represent limits of detection.

We next examined levels of vRNA in midgut, leg and wing, salivary gland, and saliva samples of infected mosquitos (Fig. 5). We found high levels of midgut WNV RNA, which was not impacted by coinfection (Fig. 5a). Conversely, SLEV RNA midgut levels were significantly reduced by WNV coinfection at 7 and 14 days post-infection (*P* < 0.05) (Fig. 5a). WNV and SLEV RNA levels in legs and wings, salivary glands, and saliva were not impacted by coinfection (Fig. 5b, c and d). When examining coexposed mosquitoes, we found that the majority (>93%) of midguts were coinfected with both viruses, with a very small number only infected with WNV (the remaining were uninfected) (Fig. 5d).

In legs and wings, salivary glands, and saliva, of infected mosquitoes, large fractions were coinfected or just WNV infected, with a smaller fraction infected with just SLEV (Fig. 5e). We compared levels of WNV and SLEV in coexposed mosquitoes to determine if there was a relationship between the levels of the two viruses within each tissue/sample (Fig. 5f). There was a positively correlated relationship between levels of WNV and SLEV RNA in the midgut (Spearman *r* = 0.77), although WNV levels were consistently slightly higher than SLEV (Fig. 5f). Within legs and wings, salivary glands, and saliva, we saw no relationship between levels of WNV and SLEV at either 7 or 14 days post-infection (Spearman *r* < 0.3). Of samples that were positive for both viruses, the pattern was highly variable, with some containing high levels of both viruses and others just containing high levels of one virus (Fig. 5f).

## DISCUSSION

While coinfection of WNV and SLEV in humans, birds, or mosquitoes is likely a rare event, the likelihood of it occurring is increasing due to the seeming rebound of SLEV in the western U.S. The impact of coinfection on many aspects of virus biology, host immune response, and transmission by mosquitoes remains poorly understood. While there are countless possible viral outcomes of coinfection, they can be broadly distilled into the following three categories: (i) competition resulting in suppression/inhibition of one or both viruses, (ii) cooperation/synergy resulting in enhancement/augmentation of one or both viruses, and (iii) neutral (no impact to either virus) (25, 45). These outcomes will be influenced not only by the replication strategies and immune evasion mechanisms of any two viruses but by the host they are infecting (at both the organismal and cellular levels) and how intracellular resources (e.g., replication machinery) and pathogen recognition (e.g., innate immune sensing and evasion) differentially recognize a single versus coinfection. Because of these and other factors, it is possible that coinfection might alter disease and pathogenesis in humans and birds and vector competence and transmission dynamics in mosquito vectors [reviewed in detail by Ciota, A.T. (45)]. It is therefore critical to study these outcomes during coinfection to better understand the potential risk of coinfection on human disease, transmission risk, and the possibility of rare variant emergence.

The literature on the extent and outcomes of virus-virus interaction during coinfection remains contradictory. While enhancement has rarely been detected, interference of one virus by another has been detected in some, but not all, systems (45). Therefore, initial studies examined whether simultaneous infection by WNV and SLEV in vertebrate cells might enhance or suppress either virus. Our results demonstrated the minimal impact of coinfection in Vero cells but significant suppression of WNV replication in chicken DF-1 cells in the presence of coinfecting SLEV, particularly at low MOI. Furthermore, we saw a high frequency of Vero cells coinfected with both WNV and SLEV. It is unclear why suppression of WNV in DF-1 cells is only seen at low MOI; however, it’s possible that WNV replicates slower than SLEV, so it may be less efficient at shielding replication complexes and replicating RNA early in infection, which could then more effectively prime relevant antiviral pathways.

The lack of widespread enhancement of virus production in coinfected cultures was not surprising given that this phenomenon is rarely reported in the literature. While we observed significant suppression of WNV by SLEV in DF-1 cells, the impact was highly context dependent and was most apparent at low MOI. This lack of consistency across our study systems perhaps explains the ambiguity in much of the literature on the impact of coinfection. Importantly, these studies were conducted using highly reductionist *in vitro* systems that clearly are not intended to recapitulate the entire virus-host interaction. Additionally, the coinfection studies reported here measured viral RNA instead of infectious virus. While this is suboptimal in many cases, in this particular situation, it was our only viable option because WNV and SLEV both generate plaques in Vero cells. SLEV requires longer for plaque formation (5 days), at which point WNV would have infected and likely killed the entire monolayer, making it impossible to detect SLEV plaques. Despite our reliance on molecular assays and not functional infectious assays, the data provided here allow us to conclude that under particular circumstances, one virus may significantly inhibit the replication of another, closely related virus.

Because we observed repression of WNV in the presence of coinfecting SLEV in avian cells, we next examined the impact of coinfection using ecologically relevant host animals, American robins (*T. migratorius*). Our results revealed that robins could become simultaneously coinfected with both viruses, generating comparable levels of viremia and neutralizing antibody responses as compared with singly infected birds. There is limited experimental coinfection of birds with multiple flaviviruses; however, chickens and turkeys have been coinfected with two avian influenza viruses, influenza and Newcastle disease virus, and influenza and infectious bronchitis virus (46–48). Importantly, WNV and USUV coinfection has been detected in multiple wild birds, including owls and gulls, suggesting coinfection with WNV and SLEV might occur (27). It will be important to study and better understand coinfection dynamics and potential virus-virus interactions in birds naturally infected with multiple flaviviruses.

We next observed coinfection in multiple *Culex* cell lines and, consistent with other studies, saw no evidence of suppression. Additionally, in both *Cx. quinquefasciatus* and *Cx. tarsalis* mosquitoes, we saw no impact of coinfection on overall infection rates or levels of viral RNA. This is consistent with many experimental infections demonstrating the minimal impact of coinfection on mosquito infection, dissemination, and transmission (38–40, 42). Within *Cx. quinquefasciatus* midguts, we saw significantly lower levels of SLEV when coinfected with WNV; however, we saw no differences between single infection and coinfection in legs and wings, salivary glands and saliva. Others have seen similar suppression between viruses during coexposure (41, 43), demonstrating that coinfection dynamics are highly variable and dependent on virus, mosquito and tissue types measured, and experimental conditions.

While there are many studies determining the role of coinfection in cells and mosquitoes, very few have evaluated the ability of individual cells to become coinfected. Brustolin et al. and Goertz et al. identified Vero and mosquito cells (Aag2 and C6/36, respectively) coinfected with MAYV and ZIKV, and CHIKV and ZIKV (38, 49). Others have looked at coinfection of unique genotypes of the same virus (poliovirus, influenza virus, etc.) within individual cells, showing that coinfection occurs (50, 51). Additionally, reassortment of multipartite viruses, such as Rift Valley fever virus and bluetongue virus, is dependent on different genotypes coinfecting individual cells, allowing segments from different parental strains to be packaged as chimeric progeny (52–54).

There are many outstanding questions regarding the potential role of viral coinfection in cells, mosquitoes, and other hosts in nature (birds, humans, etc.). While our results reveal that the viruses rarely appear to interact with one another within an organism or cell, it is possible they exert distal effects on one another. RNA viruses exist as genetically complex mutant swarms, which allow them to rapidly adapt to different hosts, temperatures, environments, etc. (e.g., alternating between vertebrate and invertebrate hosts) (55). It is possible that coinfection applies a selective pressure on one or both viruses, altering virus population diversity, complexity, or overall structure. It has been shown for multiple arboviruses that smalls changes to the genome can lead to large impacts on virus transmission, vector competence, etc. Both CHIKV and WNV acquired single coding changes in their envelope proteins resulting in increased transmission by *Ae. albopictus* mosquitoes and more efficient and faster transmission by *Culex* mosquitoes, respectively (56, 57). Coinfection might alter the likelihood or rate of rare genotypes emerging, and therefore, it is important to understand the role of coinfection of virus population structure. Therefore, it is critical to better understand these coinfection dynamics and the potential they have to impact variant emergence, human disease, and transmission risk.

## MATERIALS AND METHODS

### Cells and viruses

Vero cells (CCL-81) (African green monkey kidney) and DF-1 (chicken fibroblast) cells were maintained in DMEM supplemented with 5% fetal bovine serum (FBS) at 37°C (Vero) and 40°C (DF-1) and 5% CO_2_. CT (*Cx. tarsalis*) cells were maintained in Schneider’s media supplemented with 7% FBS at 28°C and no CO_2_. Hsu (*Cx. quinquefasciatus*) cells were maintained in DMEM with 10% FBS and 10% tryptose phosphate broth at 28°C and 5% CO_2_. All media were further supplemented with 10 units/mL penicillin, 10 µg/mL streptomycin, and 2.5 µg/mL amphotericin B. WNV strain FtC-3699 (accession #KR868734.1) and SLEV strain TVP-9083 were passaged one time on Vero cells, supernatant aliquoted, and frozen at −80°C prior to use.

### Plaque assay

Standard plaque assays were used to quantify infectious virus. Briefly, Vero cells were plated the day prior to infection, and virus was serially diluted, added to cells, and incubated at 37°C for 1 hour. Cells were overlaid with a semisolid tragacanth medium and incubated for 3 (WNV) and 5 (SLEV) days, then fixed, and stained with 20% ethanol and 0.1% crystal violet. Plaques were counted manually.

### Growth curves

Cells were plated 1–2 days prior to infection. MOIs were calculated for each virus (e.g., 0.01 MOI WNV, 0.01 MOI SLEV, or 0.01 MOI WNV and 0.01 MOI SLEV). WNV and SLEV were diluted in infection media (regular growth media with 1% FBS) and added to cells for 1 hour. Cells were washed three times with PBS, and fresh growth media were added. Supernatant was sampled daily, RNA was extracted, and qRT-PCR was performed as described below.

### RNA extraction and qRT-PCR

RNA was extracted using the MagMAX Viral Pathogen Nucleic Acid Isolation 96-well Kit on a KingFisher Flex machine according to the manufacturer’s instructions. qRT-PCR was performed using EXPRESS One-Step qRT-PCR Kits according to the manufacturer’s instructions. WNV and SLEV qRT-PCR primer probes targeting the envelope gene region were previously described (Table 3) (58, 59). WNV viral RNA standards as previously described were used to generate copy numbers (60). SLEV whole genome viral RNA was used as a standard to extrapolate copy numbers.

**TABLE 3:** WNV and SLEV primer and probe sequences

Virus	Oligo	Sequence (5′ → 3′)
WNV	Forward	TCAGCGATCTCTCCACCAAAG
	Reverse	GGGTCAGCACGTTTGTCATTG
	Probe	TGCCCGACCATGGGAGAAGCTC
SLEV	Forward	CTGGCTGTCGGAGGGATTCT
	Reverse	TAGGTCAATTGCACATCCCG
	Probe	TCTGGCGACCAGCGTGCAAGCCG

### Microscopy

Cells were infected as described in growth curves (above), using MOIs of 1 and 5 and fixed 3 (vertebrate) or 5 (mosquito) days post-infection. Infected cells were fixed in 10% buffered formalin at room temperature for at least 2 hours and then stored at 4°C until staining. Samples were permeabilized in permeabilization buffer (1× phosphate buffered saline, 1% bovine serum albumin, and 0.1% Triton X-100) for 30 minutes at room temperature and then blocked in permeabilization buffer containing 1% FBS for 30 minutes at room temperature. All samples were stained with mouse anti-SLEV NS1 antibody (EastCoast Bio #HM940) and rabbit anti-WNV capsid antibody (Genetex #GTX131947) at 1:1,000 for 1 hour at 37°C. Samples were washed in PBS buffer and then stained with secondary anti-mouse-AlexaFluor-594 (Cell Signaling #8890) and anti-rabbit-AlexaFluor-488 (Invitrogen #A11008) diluted 1:2,000 and Hoescht dye for 1 hour at 37°C. Samples were washed and imaged on a Revolve Echo Fluorescent Microscope.

### Flow cytometry

Cells were trypsinized (Vero) or scraped (CT) into a single-cell suspension, then fixed in 4% paraformaldehyde, processed, and stained as described for microscopy (above). Once stained, cells were analyzed on a Cytek Aurora four channel flow cytometer. Flow plots are shown in Fig. S2 and S5. Flow cytometry data were analyzed on FlowJo Version 10.8.1.

### America robin infections

Birds were housed in 0.5–1 m^3^ cages in groups of three to four with space for limited flight and fed *ad libitum* water and dry dog food supplemented with earth worms/meal worms as previously described (61, 62). Pre-infection, robins were bled and analyzed for pre-existing anti-WNV antibodies via standard plaque reduction neutralization test (described below). SLEV is not present in Colorado, so pre-infection serum was not screened for SLEV antibodies. Robins were subcutaneously inoculated with 10^4^ PFU WNV, 10^4^ PFU SLEV, or 10^4^ PFU WNV and 10^4^ PFU SLEV diluted in PBS in a total of 100 μL. Animals were monitored daily post-infection for any signs of disease. On days 1–5, 7, and 14 post-infection, blood was collected in a serum separator microtainer tube (BD, catalog #365967) via a jugular vein. On day 21, blood was collected from each bird, followed by euthanasia via sodium pentobarbital overdose. Blood was allowed to clot at room temperature for 30 minutes and then spun for 5 minutes at 1,200 × *g*. Serum was removed and stored at −80°C until testing for viral RNA as described above. All animal infections were conducted at Colorado State University under ABSL-3 containment.

### Neutralization assay

Serum from days 14 and 21 post-inoculation was heat inactivated at 56°C for 30 minutes and then stored at 4°C prior to neutralization assays. A standard plaque reduction neutralization tested was performed against both WNV and SLEV. Briefly, Vero cells were plated 1 day prior to infection. A dilution series of heat-inactivated serum was mixed with ~45 PFU of either WNV or SLEV and incubated for 1 hour at 37°C. The virus:serum mixture was added to confluent Vero cells, incubated for 1 hour at 37°C, then overlaid with semisolid tragacanth medium, and incubated for 3 (WNV) and 5 (SLEV) days. Cells were fixed and stained with 20% ethanol and 0.1% crystal violet, and plaques were counted manually. PRNT_50_ (plaque reduction neutralization 50 titers—the serum dilution factor required to neutralize 50% of infectious virus) were calculated in GraphPad Prism Version 9.3.1.

### Mosquito rearing

Colonies of *Cx. quinquefasciatus* (established from wild populations collected in Florida in 1988) and *Cx. tarsalis* (established from a colony maintained by WK Reisen collected in California in 1953) were maintained at 26°C–27°C with a 16:8 light:dark cycle and 70%–80% relative humidity, with water and sugar provided *ad libitum*. Larvae were raised on powdered fish food.

### Mosquito infections and dissections

In a BSL-3/ACL-3 insectary, female *Culex* mosquitoes (5–8 days post-eclosion) were fed an infectious bloodmeal containing defibrinated calf blood and virus. Concentrations of bloodmeals were ~10^7^ PFU/mL WNV, ~10^7^ PFU/mL SLEV, or ~10^7^ PFU/mL WNV and ~10^7^ PFU/mL SLEV. Bloodmeals were heated to 37°C via a water bath and fed to mosquitoes using water-jacketed glass feeders sealed with a layer of hog’s gut. Mosquitoes were fed for 1 hour and then cold anesthetized, and engorged females were sorted. Exposed mosquitoes were held for 7 or 14 days with water and sugar provided *ad libitum*. For dissections, mosquitoes were cold anesthetized, legs and wings were removed, and mosquitoes were salivated into capillary tubes containing immersion oil for 30 minutes. Salivary glands and midguts were then dissected. Midguts, legs and wings, and salivary glands were placed into tubes containing a ball bearing and mosquito diluent (PBS, 20% FBS, 50 µg/mL penicillin/streptomycin, 50 µg/mL gentamicin, and 2.5 µg/mL amphotericin B), homogenized, and then centrifuged. Capillary tubes containing saliva were placed into tubes containing mosquito diluent and centrifuged to expel saliva from capillaries. RNA was immediately extracted from samples and bloodmeals, viral RNA was measured via qRT-PCR as described above, and then, samples were stored at −80°C. Consolidated results from all mosquito experiments are provided in Table S1.

### Data analysis and statistics

All data were analyzed using GraphPad Prism Version 9.3.1. Statistical tests are described in figure legends.

## Data Availability

All data are available upon request.

## SUPPLEMENTAL MATERIAL

Supplemental figures jvi.01041-24-s0001.docx Figures S1 to S5.

Table S1 jvi.01041-24-s0002.xlsx Mosquito experimental infection metadata.

ASM does not own the copyrights to Supplemental Material that may be linked to, or accessed through, an article. The authors have granted ASM a non-exclusive, world-wide license to publish the Supplemental Material files. Please contact the corresponding author directly for reuse.
