# Supplementary material for: WNV and SLEV coinfection in avian and mosquito hosts: impact on viremia, antibody responses, and vector competence
Source: J Virol. 2024 Sep 26;98(10):e01041-24. doi: 10.1128/jvi.01041-24 (PMC11495067; doi:10.1128/jvi.01041-24)
Supplement: Supplemental figures — Figures S1 to S5. [file jvi.01041-24-s0001.docx]

**
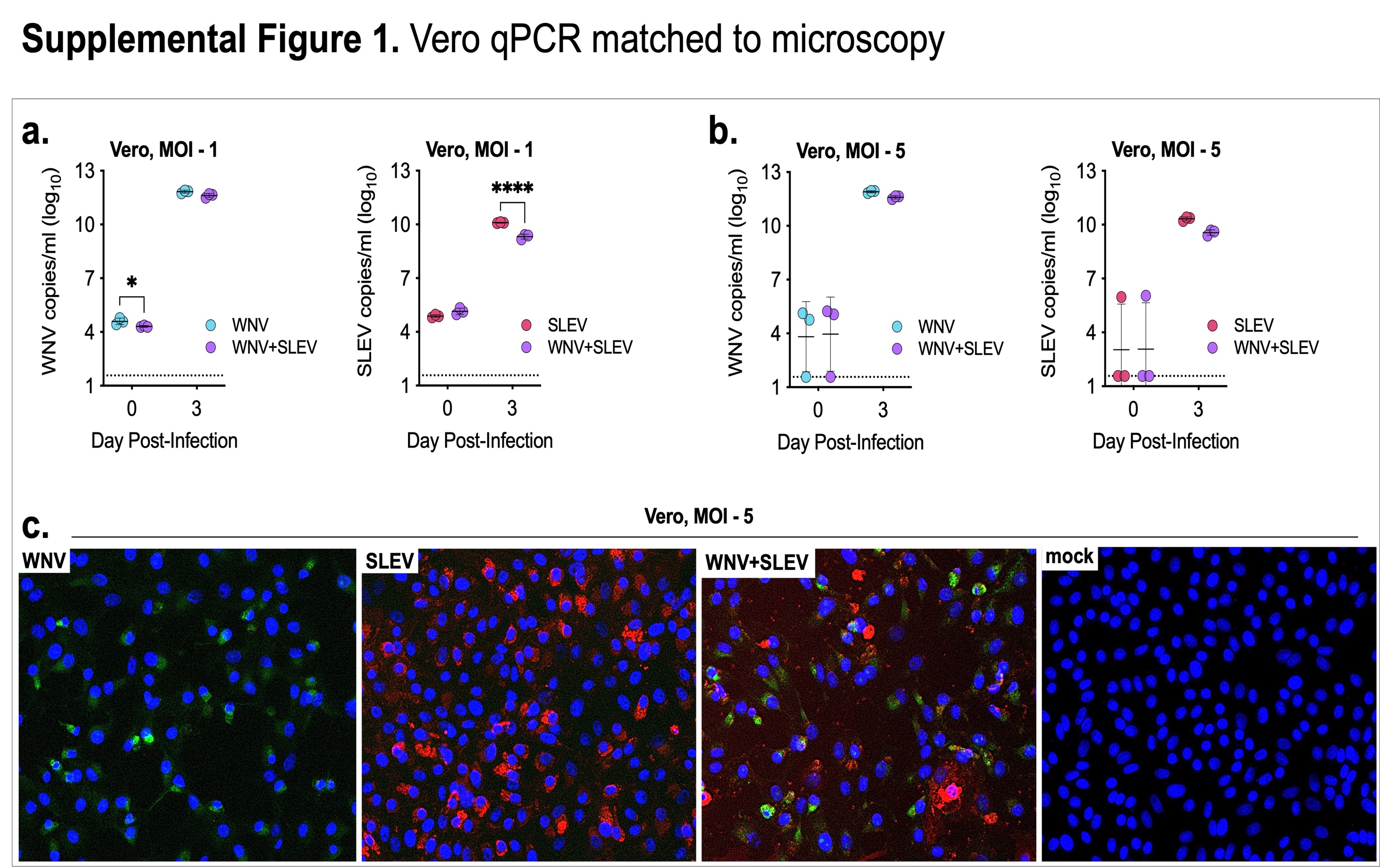
**

**Supplemental Figure 1. Vero qPCR from microscopy samples.** Vero (African green monkey) cells were infected at two multiplicities of infection (MOI) **a)** 1 and **b)** 5 individually, or coinfected with WNV and SLEV. On day 3 post-infection, supernatant was sampled and tested for viral RNA as measured by qRT-PCR (performed in biological triplicate, mean ± standard deviation). Two-way ANOVA with Šidák’s multiple comparison test (*p<0.05, ****p<0.0001). **c**) Vero cells were individually or coinfected (MOI = 5), and after 3 days fixed and stained for WNV and SLEV viral protein.

**
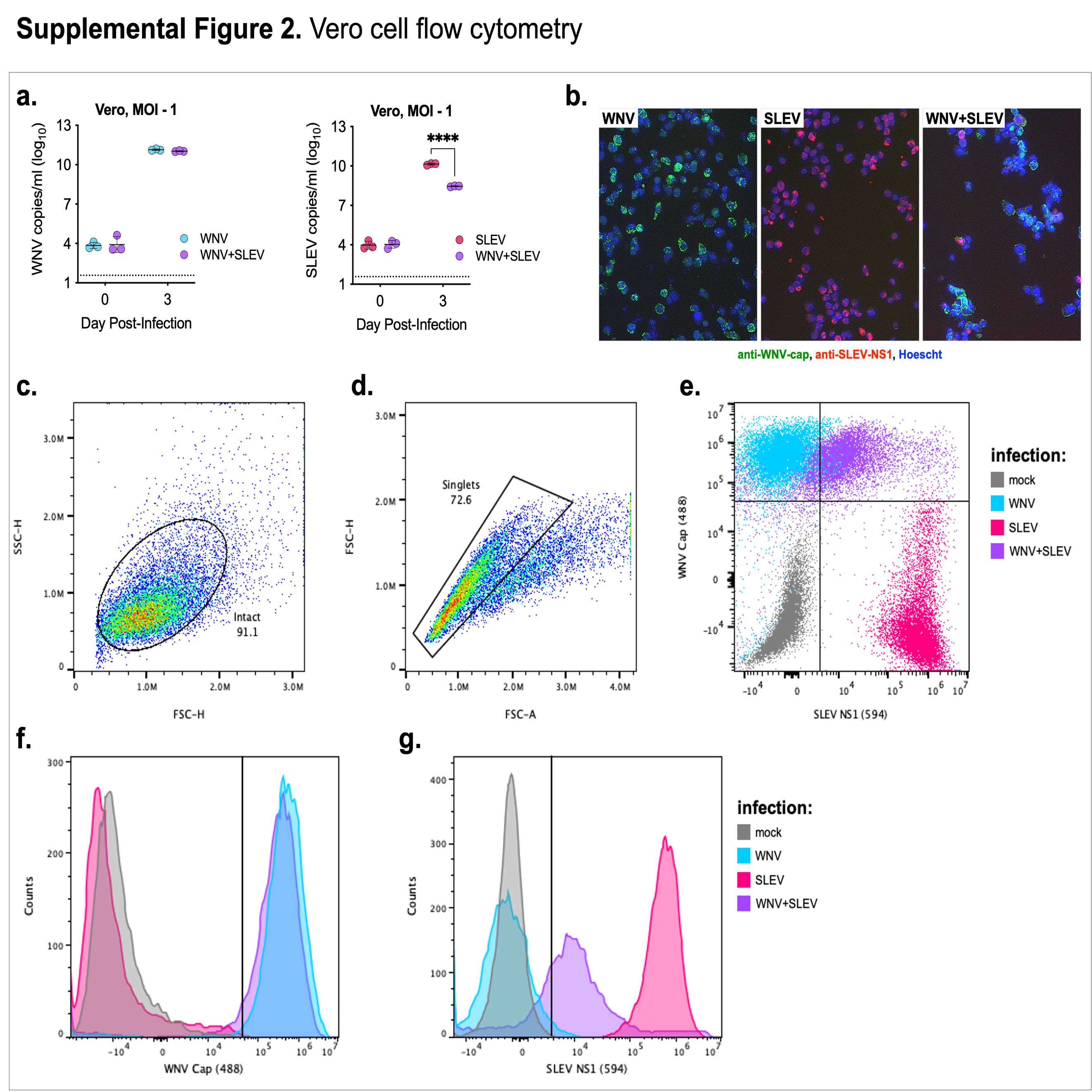
**

**Supplemental Figure 2. Vero cell flow cytometry.** Vero (African green monkey) cells were infected at a MOI of 1, individually, or coinfected with WNV and SLEV. **a)** On day 3 post-infection, supernatant was sampled and tested for viral RNA as measured by qRT-PCR (performed in biological triplicate, mean ± standard deviation). Two-way ANOVA with Šidák’s multiple comparison test (****p<0.0001). Cells were stained for viral protein for **b)** microscopy (additionally stained with Hoescht) and **c-g)** flow cytometry. **c)** Cells were first gated on forward scatter (FSC) and side-scatter (SSC) for intact cells, then **d)** forward scatter area (FSC-A) and forward scatter height (FSC-H) for singlets. **e)** Cells were then analyzed for WNV capsid (488) and SLEV NS1 (594) protein and gated into WNV positive/negative and SLEV positive/negative populations. Histogram plots of **f)** WNV capsid (488) and **g)** SLEV NS1 (594). Representative plots from a single replicate of each infection condition are shown.

**Supplemental Figure 3. Robin serum PRNT_80_ analyses. a)** Serum collected on days 14 and 21 were analyzed for neutralization against both WNV and SLEV using a standard plaque reduction neutralization test. PRNT_80_ (serum dilution factor required to neutralize 80% of virus) are plotted (mean ± standard deviation). Samples with no neutralization are plotted at half the limit of detection. Two-way ANOVA with Tukey’s multiple comparisons test (*p<0.05). **b)** Relationship between WNV and SLEV neutralization titers (diamond – day 14, square – day 21). Dashed lines represent limits of detection.

**
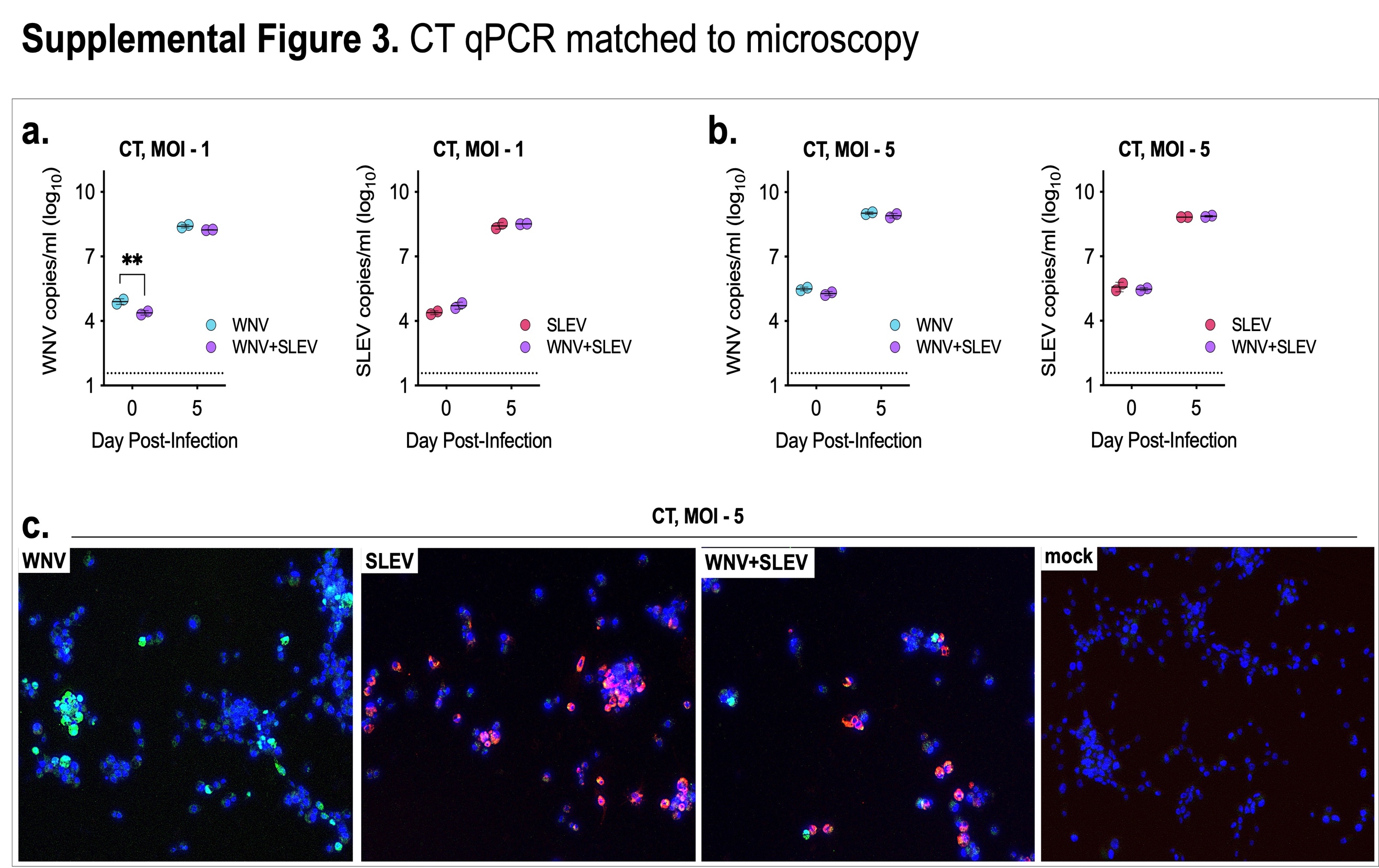
**

**Supplemental Figure 4. CT qPCR from microscopy samples.** CT (*Cx. tarsalis*) cells were infected at two multiplicities of infection (MOI) **a)** 1 and **b)** 5 individually, or coinfected with WNV and SLEV. On day 5 post-infection, supernatant was sampled and tested for viral RNA as measured by qRT-PCR (performed in biological duplicate, mean ± standard deviation). Two-way ANOVA with Šidák’s multiple comparison test (**p<0.01). **c**) CT cells were individually or coinfected (MOI = 5), and after 5 days fixed and stained for WNV and SLEV viral protein.

**
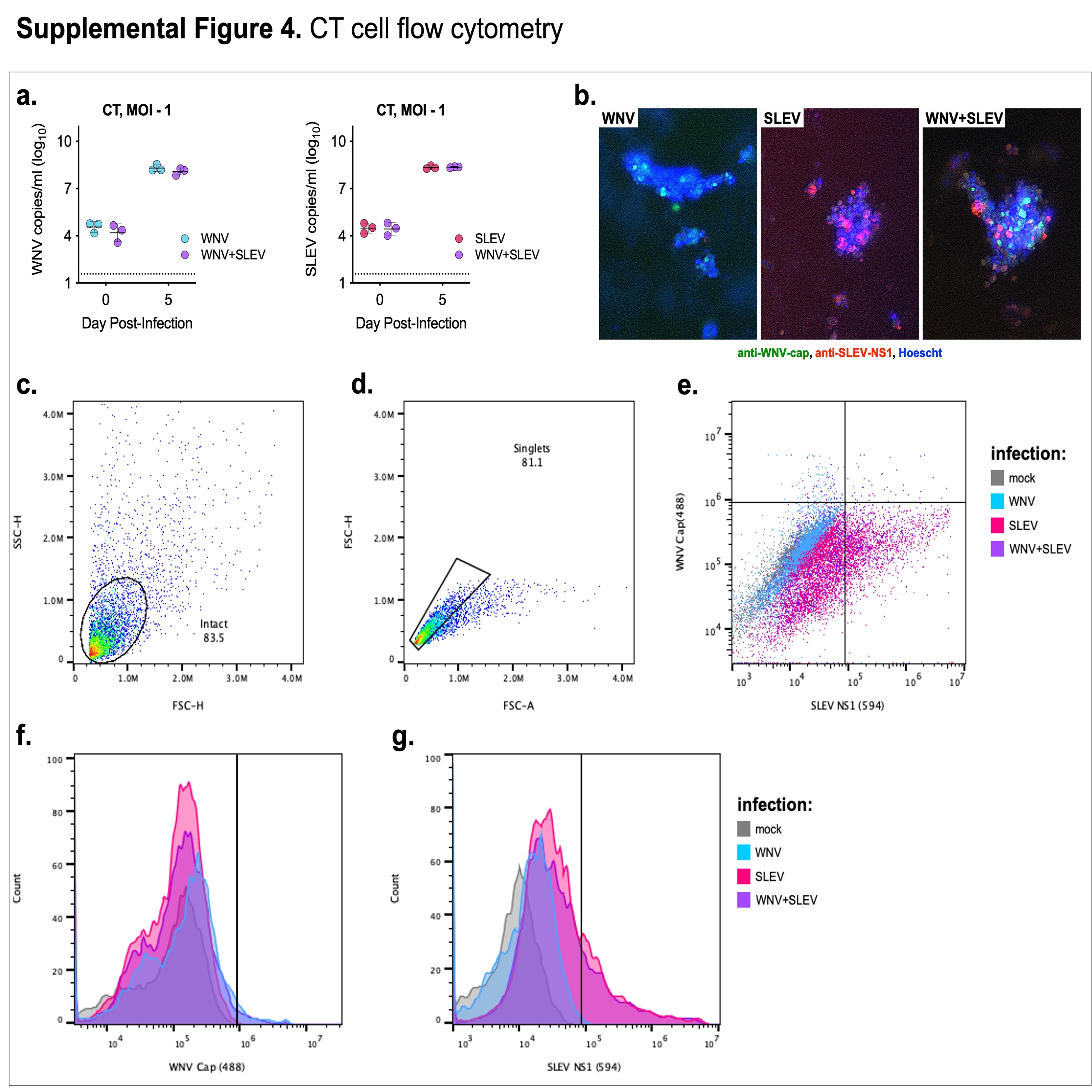
**

**Supplemental Figure 5. CT cell flow cytometry.** CT (*Cx. tarsalis*) cells were infected at a MOI of 1, individually, or coinfected with WNV and SLEV. **a)** On day 5 post-infection, supernatant was sampled and tested for viral RNA as measured by qRT-PCR (performed in biological triplicate, mean ± standard deviation). No comparisons were significant using two-way ANOVA with Šidák’s multiple comparison test (p>0.05). Cells were stained for viral protein for **b)** microscopy (additionally stained with Hoescht) and **c-g)** flow cytometry. **c)** Cells were first gated on forward scatter (FSC) and side-scatter (SSC) for intact cells, then **d)** forward scatter area (FSC-A) and forward scatter height (FSC-H) for singlets. **e)** Cells were then analyzed for WNV capsid (488) and SLEV NS1 (594) protein and gated into WNV positive/negative and SLEV positive/negative populations. Histogram plots of **f)** WNV capsid (488) and **g)** SLEV NS1 (594). Representative plots from a single replicate of each infection condition are shown.
